# Improved CSW-YOLO Model for Bitter Melon Phenotype Detection

**DOI:** 10.3390/plants13233329

**Published:** 2024-11-27

**Authors:** Haobin Xu, Xianhua Zhang, Weilin Shen, Zhiqiang Lin, Shuang Liu, Qi Jia, Honglong Li, Jingyuan Zheng, Fenglin Zhong

**Affiliations:** 1 College of Horticulture, Fujian Agriculture and Forestry University, Fuzhou 350002, China; 52303030056@fafu.edu.cn (H.X.); zxh850925835@126.com (X.Z.); 15206008553@163.com (W.S.); liush@fafu.edu.cn (S.L.); 2 Institute of Vegetables, Hunan Academy of Agricultural Sciences, Changsha 410125, China; 3 Jiuquan Academy of Agriculture Sciences, Jiuquan 735099, China; jq17793520924@163.com; 4 Fujian Agricultural Machinery Extension Station, Fuzhou 350002, China; zhiking@126.com; 5 Fujian Tianmei Seed Industry Technology Co., Fuzhou 350109, China; 13805006170@139.com

**Keywords:** bitter melon, phenotypic detection, deep learning, CSW-YOLO

## Abstract

As a crop with significant medicinal value and nutritional components, the market demand for bitter melon continues to grow. The diversity of bitter melon shapes has a direct impact on its market acceptance and consumer preferences, making precise identification of bitter melon germplasm resources crucial for breeding work. To address the limitations of time-consuming and less accurate traditional manual identification methods, there is a need to enhance the automation and intelligence of bitter melon phenotype detection. This study developed a bitter melon phenotype detection model named CSW-YOLO. By incorporating the ConvNeXt V2 module to replace the backbone network of YOLOv8, the model’s focus on critical target features is enhanced. Additionally, the SimAM attention mechanism was introduced to compute attention weights for neurons without increasing the parameter count, further enhancing the model’s recognition accuracy. Finally, WIoUv3 was introduced as the bounding box loss function to improve the model’s convergence speed and positioning capabilities. The model was trained and tested on a bitter melon image dataset, achieving a precision of 94.6%, a recall of 80.6%, a mAP50 of 96.7%, and an F1 score of 87.04%. These results represent improvements of 8.5%, 0.4%, 11.1%, and 4% in precision, recall, mAP50, and F1 score, respectively, over the original YOLOv8 model. Furthermore, the effectiveness of the improvements was validated through heatmap analysis and ablation experiments, demonstrating that the CSW-YOLO model can more accurately focus on target features, reduce false detection rates, and enhance generalization capabilities. Comparative tests with various mainstream deep learning models also proved the superior performance of CSW-YOLO in bitter melon phenotype detection tasks. This research provides an accurate and reliable method for bitter melon phenotype identification and also offers technical support for the visual detection technologies of other agricultural products.

## 1. Introduction

Bitter melon (*Momordica charantia* L.) is an annual herbaceous plant of the Cucurbitaceae family and an important economic crop with both medicinal and culinary uses. Due to its unique medicinal value and nutritional components, it is widely favored by consumers [1] and is now extensively cultivated from the tropics to the temperate zones worldwide. Not only is bitter melon a highly nutritious vegetable, but it also has significant medicinal value and is widely used in traditional medicine [2]. It contains a variety of vitamins, minerals, and dietary fibers, offering health benefits such as cooling the body, reducing blood sugar levels, and lowering blood lipids. Driven by the global trend towards healthy eating, the demand for bitter melon continues to grow, particularly in Asia and Africa, making it an important crop in the vegetable industry [3]. As consumer interest in healthy diets increases, the demand for bitter melon continues to rise, especially among young people and those with a strong health consciousness. The diversity of shapes in bitter melon is one of its important genetic characteristics, directly affecting the fruit’s market acceptance and consumer preferences [4]. Therefore, breeding more high-quality new varieties has become an urgent need for the development of the bitter melon industry. Precise, intelligent, and high-throughput identification of bitter melon germplasm resources is fundamental to the successful execution of genetic breeding work. Additionally, phenotypic identification of large populations during the breeding process is one of the most critical steps in breeding. However, most phenotypic identifications still rely on manual methods, which are not only time-consuming and labor-intensive but also susceptible to subjective factors, making accuracy difficult to guarantee. Therefore, developing automated and intelligent bitter melon phenotypic identification technology is a critical direction for the industry’s development.

With the increasing demand for plant phenotypic identification, there has been a push for the application of computer image processing and deep learning techniques in agriculture, particularly in fruit detection and classification [5,6]. Deep learning methods, utilizing convolutional neural networks, can extract rich shallow features and deep semantic features, gradually replacing traditional image processing algorithms. They are favored for their speed and high precision in practical applications [7,8]. In existing research, numerous scholars have applied their excellent image recognition capabilities across multiple fields. For example, Aich et al. [9] used deep convolutional and deconvolutional networks to segment rosette plant areas and count leaves, thereby providing strong support for plant phenotypic research. Wang Shoufu et al. [10] proposed a WPA-SVM succulent plant classification model that takes composite features of color and texture as input, achieving an accuracy rate and misclassification rate of 99.42% and 0.58%, respectively, on an original dataset of five succulent plant classes. Zhao Zhiyan et al. [11] developed a convolutional neural network pest identification model that identified three types of pests—gold beetles, pear psyllids, and pear gall midges—in 1013 images of pear leaves, with an accuracy rate of 81.18%. Tong Zhen et al. [12] combined the characteristics of ResNet and UNet to segment tree images in natural scenes, using ResNet34 for feature extraction and UNet for upsampling, fully exploiting the semantic relationships of image pixels. Lobo-Torres et al. [13] used DeepLabv3+, based on MobileNetv2 as the backbone network, to segment endangered tree species from drone-captured forest overhead views, achieving an accuracy of 93.5%. Song Huaibo et al. [14] used the YOLOv5s model to accurately locate camellia fruits in complex natural scenes. Farjon et al. [15] proposed a flower detector based on deep convolutional neural networks, used for detecting blooming, with an average accuracy of 68%. Bai et al. [16] proposed an improved YOLO algorithm with a Swin-Transformer prediction head built on high-resolution feature maps, capable of rapidly and accurately recognizing strawberries, with an average precision of 92.10%. Sun Gonglingyun et al. [17] proposed a lightweight convolutional neural network based on an improved MobileNet V3 and transfer learning, achieving an accuracy of 97.35% in identifying 13 species of succulent plants. Lu J et al. [18] improved the yolov5s model, reducing model memory and parameters while enhancing the accuracy of detecting green citrus fruits in complex natural environments. Firozeh Solimani et al. [19] integrated the SE attention mechanism into the head architecture of YOLOv8, effectively improving the model’s ability to recognize tomato nodes, fruits, and flowers. Qianhui Liu et al. [20] replaced the residual network of YOLOX’s backbone feature extraction network with the bottle2neck_se module, improving the model’s recognition accuracy for uncracked small cotton bolls by 1.15%. Wendong Niu et al. [21] proposed an improved YOLOv8-ECFS weed identification model, achieving accurate identification and classification of different weeds.

Due to the high variability in the shapes of bitter melon and various environmental disturbances such as different lighting conditions and background noise, existing models often struggle to achieve the high precision and real-time performance needed in agricultural production [22]. To address these issues, this study focuses on the shapes of the top and tail of bitter melons, improving the YOLOv8 model to enhance the precision of fruit trait detection. The main contributions are as follows:

(1) First, detailed shape classification and data annotation of bitter melon fruits were carried out to establish a comprehensive bitter melon image dataset.
(2) On the basis of YOLOv8, the ConvNeXt V2 module was introduced to modify the backbone network, enhancing the model’s ability to capture features.
(3) Added the SimAM attention mechanism, which refines features further by computing attention weights through neurons.
(4) WIoUv3 was used as the bounding box loss function, improving the model’s localization performance and generalizability.

This research offers an automated and intelligent phenotypic detection technology for bitter melon, which can enhance breeding efficiency, reduce costs, and improve the precision and reliability of data, also providing technical support for the visual detection of other agricultural products.

## 2. Materials and Methods

### 2.1. Data Collection and Dataset Construction

#### 2.1.1. Data Collection

**Figure 1:**
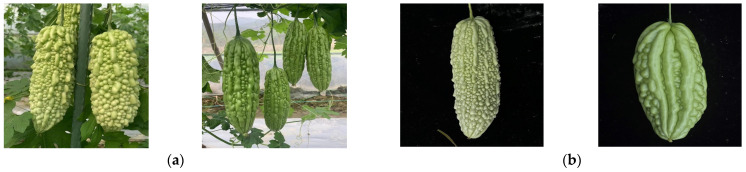
Bitter gourd images in different states: (**a**) before harvesting, (**b**) after harvesting.

The samples for this study were sourced from the Baisha Plantation Base of Tianmei Agriculture in Fujian Province, China. The bitter gourd varieties involved include the following: Yuchuan No. 2, Qisheng 308, and Tianmei 20. The image samples collected fall into two main categories: one is unharvested, with the fruit still hanging on the vine for photography (Figure 1a), and the other is of fruit that has been harvested (Figure 1b).

In this study, images were captured using smartphones, specifically the iPhone 13, iPhone 13 Pro, Redmi K50, and Redmi Note 10 Pro, with resolutions of 4032 × 3024, 4000 × 3000, and 4624 × 3472 pixels. All images were collected under natural light conditions, at a distance of 0.3–1 m, and saved in .jpg format. In total, 871 original images were obtained.

#### 2.1.2. Dataset Construction

Data augmentation is a commonly used technique that increases the sufficiency and diversity of training data by applying various transformations to the training data [23]. This not only enables the model to learn more diverse features and adapt to a wider range of input data but also helps the model learn to ignore irrelevant features and focus on more generalizable features [24].

**Figure 2:**
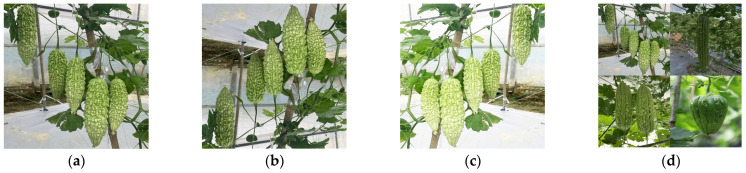
Effects of image enhancement. (**a**) Original image, (**b**) vertical flip, (**c**) mirror flip + brightness increase, (**d**) combination of multiple methods.

To prevent model overfitting and ensure that the model learns robust features under various transformations, ensuring accurate predictions when faced with new, unseen data [25], in this study, data augmentation techniques such as mirroring, brightness adjustment, Gaussian blur, contrast adjustment, random translation, and image stitching were randomly combined [26] to expand the original image data (Figure 2), resulting in a total of 2571 sample images.

Based on the differences in the shape of the top and tail of the bitter melon, and after analyzing the collected images, this study defined 12 categories, with label names, specific descriptions, and sample quantities shown in Table 1. The bitter melon images were manually annotated using the labeling tool LabelImg (v1.8.1). During annotation, it was ensured that the target was completely within the frame with a small boundary-to-target distance, and multiple targets sharing one frame were not allowed. The annotated images were randomly shuffled and then divided into a training set and a validation set at a ratio of 4:1. In this process, it is ensured that both the training and testing sets evenly contain various types of samples.

### 2.2. Improved Network Architecture

This study performed preliminary model training using several newer versions of YOLO. As shown in Table 2, YOLOv8n outperforms other versions of YOLO in all metrics on the bitter melon dataset.

**Figure 3:**
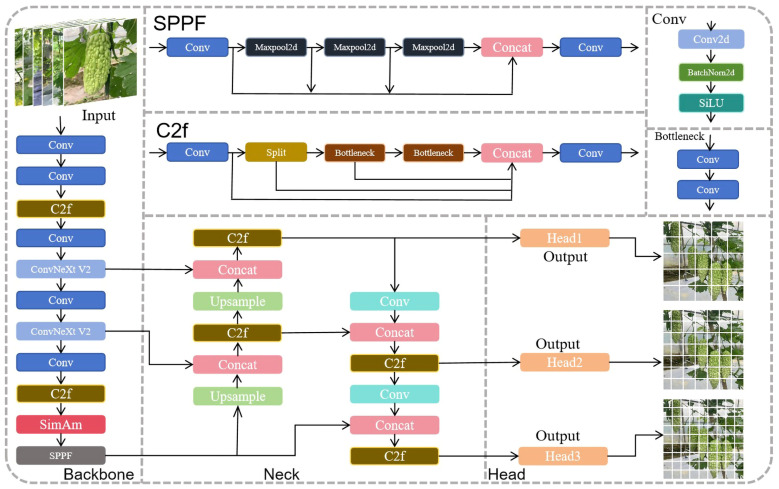
Improved network structure.

This study adopted the YOLOv8 object detection algorithm for improvement experiments, with its structure divided into three parts: the backbone network, the neck network, and the detection head [27]. The backbone network continues to use the CSP concept, utilizing the Darknet53 architecture, which includes basic convolution units (Conv) and Spatial Pyramid Pooling Forward (SPPF) to achieve local and global feature fusion. The neck network uses a PAN-FPN structure, and both it and the backbone network incorporate the gradient-enriched C2f module to merge feature maps of different sizes, enhancing network depth and field of view, further achieving lightweight design [28]. The detection head employs a decoupled head structure, separating classification and detection tasks, and it utilizes an anchor-free mechanism during detection. The loss function calculation uses the Task Aligned Assigner positive sample distribution strategy, combining classification loss VFL (varifocal loss) with regression loss CIOU (complete-IOU) and DFL (deep feature loss) in a ternary weighted combination [29]. To further enhance the precision and recall of the bitter gourd top and tail shape recognition model, this study proposes an improved network based on YOLOv8, named CSW-YOLO. The specific improvements include replacing the C2f modules in the 5th and 7th layers of the backbone network with ConvNeXt V2 modules, adding a SimAM attention mechanism (simple attention mechanism) above the SPPF layer, as well as using WIOUv3 (Wise-IoU v3) loss function to replace the original CIOU loss function (Figure 3).

#### 2.2.1. ConvNeXt V2 Module

**Figure 4:**
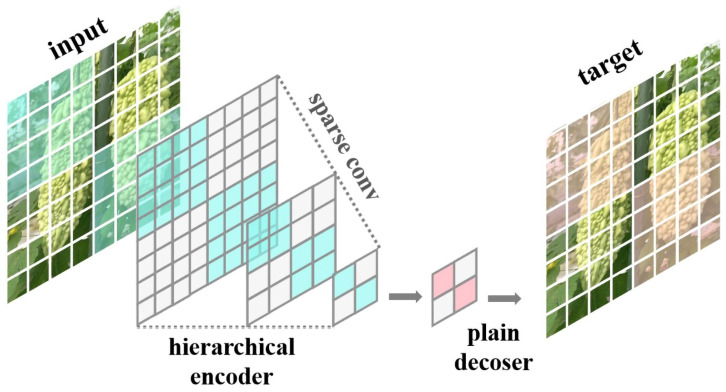
FCMAE: fully convolutional masked autoencoder.

**Figure 5:**
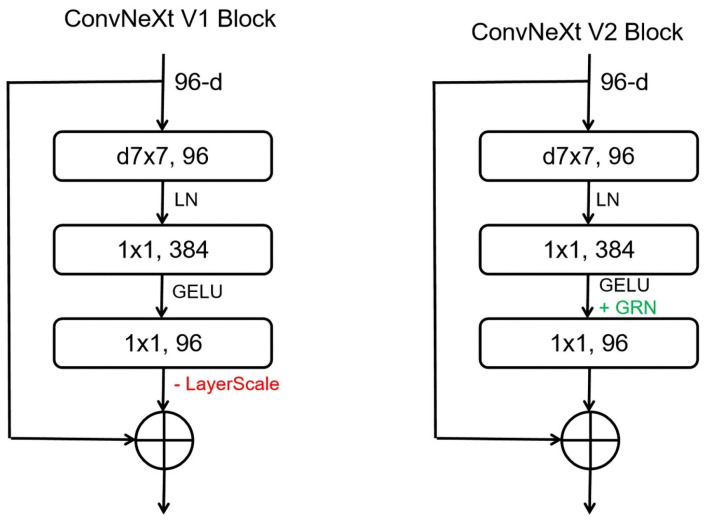
ConvNeXt block designs.

ConvNeXt V2 is a new type of convolutional neural network architecture proposed by Sanghyun Woo and others [30]. To enhance self-supervised learning effectiveness, ConvNeXt V2 employs a fully convolutional masked autoencoder (FCMAE) framework. As shown in Figure 4, the encoder part uses sparse convolution to process inputs containing only visible parts, thus reducing the computational cost of pre-training, as well as allowing the model to use the remaining context information to predict missing parts, thereby enhancing its ability to learn and understand visual data [31]. Moreover, the ConNeXt V2 model removes the LayerScale layer from the ConNeXt V1 and adds a Global Response Normalization (GRN) layer to address feature variations (Figure 5). The GRN layer can increase the contrast between channels, preventing feature collapse during the learning process [32], thus effectively enhancing model performance.

ConvNeXt V2 randomly masks parts of the bitter gourd images and then uses sparse convolution to predict the masked areas, capturing the details of the bitter gourd images, improving accurate feature capture, and reducing computational costs without sacrificing performance [33]. Additionally, the GRN layer enhances competition between feature channels, helping the model better distinguish subtle differences between bitter gourds, thereby improving recognition precision and generalization ability [34].

#### 2.2.2. SimAM Attention Mechanism

**Figure 6:**
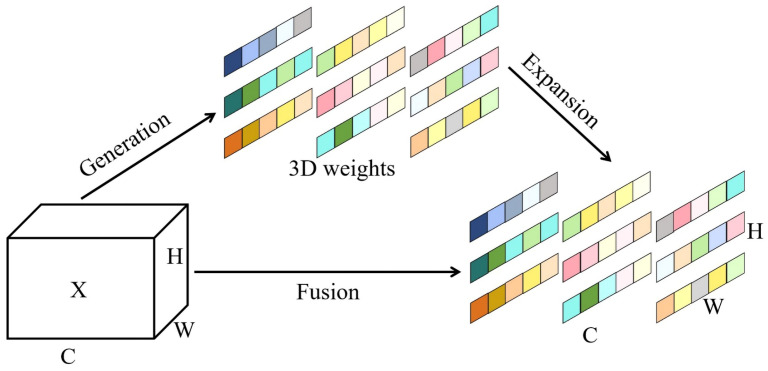
Schematic of the SimAM attention mechanism.

The attention mechanism enables neural networks to focus on the most important features in the input data, ignoring less relevant information such as background, thereby increasing the model’s sensitivity to crucial information [35]. To enhance the model’s ability to learn both deep and shallow features and make more effective use of the important features [36], this study introduces the SimAM attention mechanism into the backbone network, thus improving the overall detection precision of the model. Unlike other attention modules that refine features along channel or spatial dimensions only, SimAM can infer three-dimensional (3-D) attention weights for each neuron in the feature map without adding any parameters to the original network [37], as shown in Figure 6.

SimAM offers a parameter-free three-dimensional attention solution by calculating the importance of neurons through an energy function, which in turn calculates attention weights based on the importance of neurons to further refine features [38]. The energy function is as follows:

(1) $$e_{t}\left(w,b,y,x_{i}\right)={\left(y_{t}-â\right)}^{2}+\frac{1}{M-1}\sum_{i=1}^{M-1}{\left(y_{0}-{ẋ}_{i}\right)}^{2}$$

In this function, â = wt + b and ${ẋ}_{i}$ = wx_i_ + b are linear transformations of the target neuron and other neurons; y_t_ and y_0_ are two different values; M is the total number of neurons; and w and b are the weight and bias of the linear transformations, respectively. By minimizing this energy function, the linear separability between each neuron and its neighbors can be quantified, thus inferring the importance of each neuron.

#### 2.2.3. Loss Function

YOLOv8 employs CIoU Loss as the bounding box loss function, primarily relying on the aggregation of bounding box regression, yet the issue of misalignment between the desired true boxes and predicted boxes is overlooked [39]. Consequently, this study introduces WIoUv3, proposed by Zanjia Tong et al. [40], to replace the CIoU loss function, addressing the issue of delayed positioning of prediction boxes during training, thereby enhancing the model’s convergence speed and localization capability.

**Figure 7:**
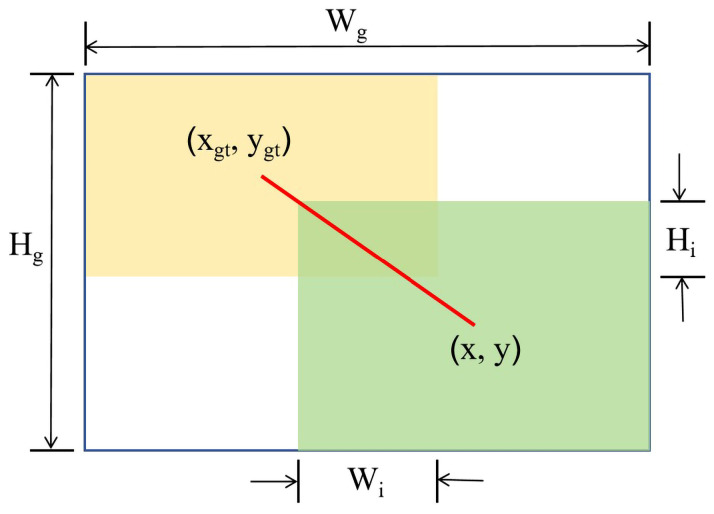
Schematic of anchor boxes and target boxes.

WIoUv3 (Wise-IoU v3) is a bounding box regression loss function based on IoU (intersection over union), incorporating a dynamic non-monotonic focusing mechanism. This mechanism dynamically adjusts gradient gains based on the quality of samples in the training data, thus reducing the negative impact of low-quality samples on model training [41]. WIoUv3 assigns gradient gains by evaluating the outlier degree of anchor boxes, allocating smaller gradient gains to high-quality anchor boxes (i.e., those with a high degree of overlap with the target box) and larger gradient gains to ordinary quality anchor boxes [42]. This method effectively mitigates the impact of low-quality samples on bounding box regression (BBR) while enhancing the model’s focus on ordinary quality samples, thereby improving the model’s localization performance and generalization capability. For anchor boxes B = {x, y, w, h} and target boxes B_gt_ = {x_gt_, y_gt_, w_gt_, h_gt_}, where the values correspond to the center coordinates and sizes of their respective bounding boxes, the location areas of the anchor and target boxes are assumed as shown in Figure 7.

The specific formula for WIoUv3 is as follows:

(2) $$L_{WIoUv3}=r\cdot R_{WIoU}L_{IoU}$$

(3) $$L_{IoU}=1-IoU=1-\frac{W_{i}H_{i}}{wh+w_{gt}h_{gt}-W_{i}H_{i}}$$

(4) $$R_{WIoU}=exp\left(\frac{{\left(x-x_{gt}\right)}^{2}+{\left(y-y_{gt}\right)}^{2}}{{\left(W_{g}^{2}+H_{g}^{2}\right)}^{*}}\right)$$

(5) $$r=\frac{\beta }{\delta \cdot \alpha^{\beta -\delta }}$$

(6) $$\beta =\frac{L_{IoU}}{\bar{L_{IoU}}}\in [0,+\infty )$$

Here, $R_{WIoU}$ denotes a runtime weight that dynamically adjusts the influence of various types of prediction boxes within the WIoU loss, optimizing the model’s error penalty distribution and enhancing overall detection accuracy [43]. W_g_ and H_g_ represent the minimum dimensions of the area enclosed by the anchor and target boxes; * denotes that the calculations of W_g_ and H_g_ are detached from the computation graph, preventing $R_{WIoU}$ from generating gradients that impede convergence; and W_i_ and H_i_ denote the dimensions of the intersecting area between the anchor and target boxes [44]. r is the gain coefficient for non-monotonic focusing, affecting the sensitivity of gradient gains to changes in $L_{IoU}$. δ and α are hyperparameters that control r and β, influencing the strategy for gradient gain distribution across prediction boxes of different quality levels.

### 2.3. Evaluation Metrics

To evaluate the performance of the CSW-YOLO model, the metrics used include precision, recall, F1 score, and mAP50, which is the mean of average precision at a threshold of 50%. The formula is as follows:

(7) $$Precision=\frac{TP}{TP+FP}\times 100\%$$

(8) $$Recall=\frac{TP}{TP+FN}\times 100\%$$

(9) $$F1=\frac{2*Precision*Recall}{\left(Precision+Recall\right)}\times 100\%$$

(10) $$AP=\frac{\sum_{1}^{k}P\times R}{K}\times 1$$

(11) $$mAP=\frac{\sum_{1}^{k}AP}{k}\times 1$$

Here, TP (true positive) represents the correct identification of the top and tail shapes of bitter melons, FP (false positive) represents the incorrect identification of these shapes, and FN (false negative) denotes the number of times the current bitter melon images are incorrectly classified as belonging to other categories.

## 3. Experimental Setup and Results Analysis

### 3.1. Experimental Environment and Hyperparameter Settings

All training and testing in this study were conducted on the same machine, equipped with an Intel Core i7-13700K CPU @ 3.4 GHz, 32 GB DDR5 RAM, and a GeForce RTX 4070 Ti GPU with 12 GB VRAM. The operating system is Windows 10 (64-bit), with CUDA 10.2 and PyTorch 1.10.1 as the deep learning framework, and Python 3.8.18 as the programming language.

In this experiment, the input size was set to 640 × 640 images, with an initial learning rate of 0.001. A momentum decay strategy was employed, set at 0.937, with a weight decay of 0.0005. The batch size per training session was set to 16.

### 3.2. CAW-YOLO Model Testing

**Figure 8:**
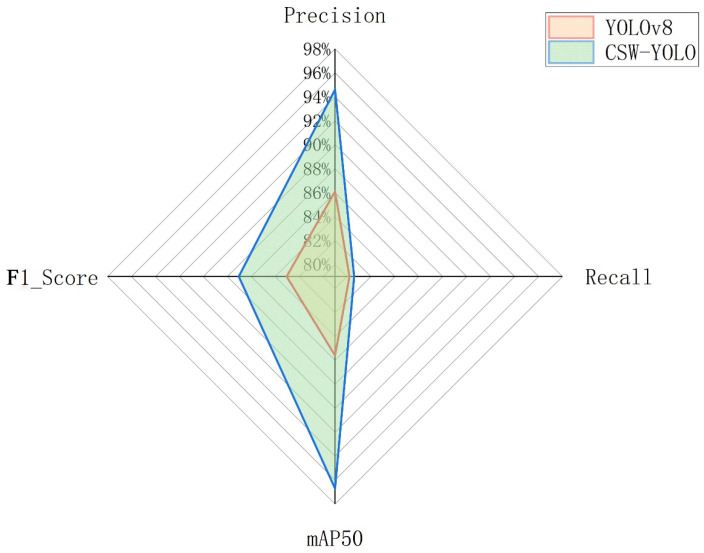
Performance comparison before and after network improvements.

To validate the performance of the CSW-YOLO model, 516 images from the test set were evaluated. The precision of the algorithm was 94.6%, recall was 80.6%, mAP50 was 96.7%, and the F1 score was 87.04%, which represents an improvement over the original YOLOv8 of 8.5%, 0.4%, 11.1%, and 4%, respectively (Figure 8).

The key to object detection lies in feature extraction. Given the constraints on the interpretability of neural network computations, to visually inspect changes in the model’s feature extraction capability due to the improvements outlined in this paper, this study employed Grad-CAM to generate heatmaps, which are reflected by generating class activation maps for the detection model [45]. In the heatmaps, the redder an area, the greater its contribution to detection [46].

**Figure 9:**
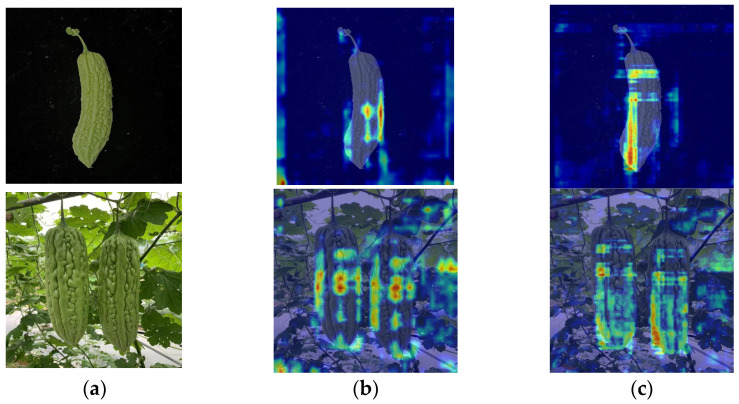
Heatmaps before and after network improvement. (**a**) Original image, (**b**) before improvement, (**c**) after improvement.

For the original YOLOv8 model, it is clear from Figure 9b that there are many hotspots outside the target area. This indicates that the network structure pays attention to some irrelevant features, which negatively impacts the model’s detection capabilities [47]. After the network modifications, the hotspots outside the target area significantly reduced or even disappeared, focusing the heatmap more on the target area (Figure 9c). This indicates that the improved CAW-YOLO model has more focused “attention” in feature extraction and is effectively reducing the attention paid to irrelevant information.

### 3.3. Ablation Study

To further verify the impact of improvements on various parts of the model, the improved algorithm of CSW-YOLO was gradually compared with the initial algorithm. The specific experimental effects are shown in Table 3, where “-” indicates that the original structure is maintained unchanged.

The results show that improvements in each part have brought performance enhancements to the model. Among them, the most noticeable improvement in performance is the introduction of the ConvNeXt V2 module in the backbone network, where all indicators significantly improved, especially the precision and mAP50, which increased by 7.4% and 11.6%, respectively. This indicates that the use of ConvNeXt V2 greatly enhances the network’s ability to capture key features. The introduction of the WIoUv3 loss function is most evident in improving recall, indicating that WIoUv3 effectively improves the network’s handling of bounding box regression, allowing the model to more comprehensively identify objects in the image. Especially when ConvNeXt V2 and WIoUv3 were combined, the recall saw the greatest improvement, from 85.60% to 97.1%, effectively reducing the model’s miss rate. The introduction of SimAM significantly improved precision, mAP50, and F1 score, with improvements in precision also surpassing the WIoUv3 loss function. However, there was a slight decrease in recall, indicating that SimAM improved the ability to discriminate key areas in the image and increased the model’s confidence when determining targets. This operation sacrificed a small part of the recall but made the target recognition more precise. Subsequent combinations show that the ConvNeXt V2 module and WIoUv3 loss function can effectively compensate for the recall loss caused by the precision improvement brought by SimAM. Finally, combining ConvNeXt V2, SimAM, and WIoUv3, the model improved in terms of precision, recall, mAP50, and F1 score, especially in precision and mAP50, which increased by 8.5% and 11.1%, respectively, indicating that the improvements in this study can effectively reduce the model’s false detection rate and accurately determine the position and size of the target while correctly identifying it. The results from the test set also show that the model can accurately identify bitter gourd in different backgrounds. In summary, the CSW-YOLO proposed in this study not only enhances the model’s precision in identifying targets but also strengthens the model’s adaptability under various backgrounds conditions.

### 3.4. Comparative Experiment

To further verify and evaluate the detection effect of the network structure proposed in this study on bitter gourd images, this study used the same dataset and operating environment to test and compare the CSW-YOLO algorithm model with mainstream models such as YOLOv7, YOLOv7-Tiny, YOLOv5, YOLOv5s, YOLOv3-tiny, Faster-RCNN, YOLOv9m, YOLOv10s, YOLOv11s, and the original YOLOv8n.

As seen from the results in Table 4, YOLOv5 had the highest recall rate and the smallest model size, at 88.70% and 3.76 M, respectively. Although CSW-YOLO lacks advantages in recall and model size, it excels in precision, mAP50, and F1 score, reaching 94.6%, 96.7%, and 87.04%, respectively, indicating that CSW-YOLO is more accurate and reliable in discriminating the shapes of the top and tail of bitter gourds. In terms of frame rate, CSW-YOLO is mid-range, but its 135.14 FPS is much higher than the 30 FPS required for real-time detection, which is sufficient to meet the needs of real-time detection.

**Figure 10:**
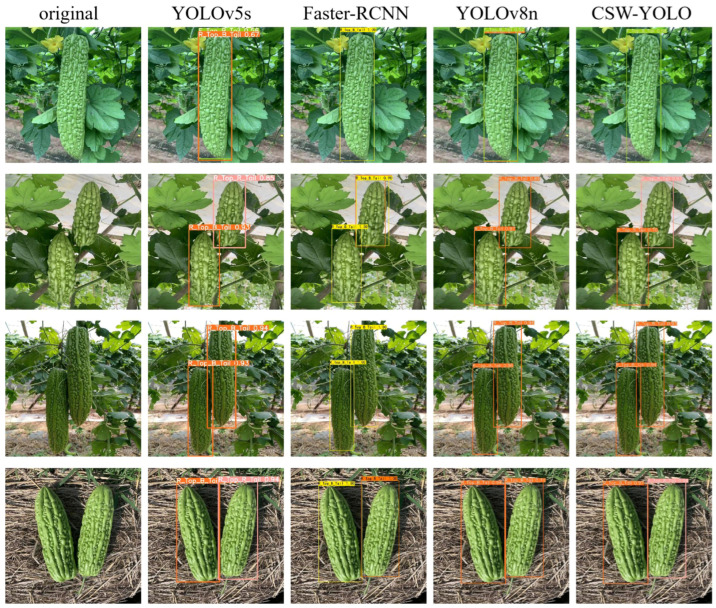
Discrimination results of different models.

Furthermore, this study also tested the detection effects of different models on the top and tail shapes of bitter gourds, with some detection results shown in Figure 10. From the results, it can be seen that CSW-YOLO significantly outperformed other models in reducing false positives and increasing accuracy. Models such as YOLOv5, YOLOv5s, YOLOv3-Tiny, YOLOv11s, and YOLOv8 had more cases of repeated detection, which not only might reduce the overall precision of the model but also increase the detection time. Faster-RCNN, YOLOv7, YOLOv7-Tiny, and YOLOv9m showed a higher number of false detections, and YOLOv7 incorrectly identified leaves as fruits, which might prevent the model from being effectively used in practice. Through comparative experiments, the CSW-YOLO model proposed in this study demonstrated superior overall performance, effectively improving the precision and reliability of detecting the shapes of bitter gourds.

## 4. Discussions

Efficient and accurate automatic detection of plant phenotypes can not only help agricultural breeders accelerate the speed of crop phenotype identification but also reduce errors and considerable time costs caused by human subjective factors [48]. Furthermore, the precise automated identification of crop phenotypes can be applied to post-harvest grading and sorting of crops, enhancing production efficiency and fruit quality management, while reducing labor costs and errors [49]. Achieving accurate detection and classification of plant phenotypes will directly impact the sustainability and economic benefits of agricultural production. By reducing labor costs and improving detection precision and efficiency, it can not only help researchers accelerate the speed of variety selection but also increase farmers’ income and provide consumers with higher quality agricultural products [50].

In this study, an improved version of the YOLOv8 model named CSW-YOLO was proposed for detecting the top and tail shapes of bitter gourds. This model has shown significant improvements in several key indicators: precision of 94.6%, recall of 80.6%, mAP50 of 96.7%, and an F1 score of 87.04%. Compared to the original YOLOv8, it increased by 8.5%, 0.4%, 11.1%, and 4% respectively. The model can better capture key features in images, reducing background interference in model discrimination, which is crucial for accurately identifying different shapes of bitter gourds in complex agricultural environments.

However, given the complexity of agricultural production conditions, the model may still face some challenges in practical applications. For example, under natural conditions such as extreme weather, the accuracy and stability of the model may be affected. Although the model in this study performs well in detecting the shape of bitter gourds, its generality and applicability to other types of fruits or crops still need further validation.

In future work, we will further test and optimize the model in the following aspects: Increase the number of bitter gourd images under extreme illumination and more complex background conditions to enhance the model’s robustness and adaptability. Explore the use of more advanced image preprocessing and enhancement techniques, such as illumination normalization and background reduction, to reduce the impact of environmental factors on detection performance [51]. Develop and find different algorithms to perfect other modules of the model, further enhancing its ability to understand complex image features. We hope that through further research and practice, we can provide reliable technical support and reference methods for the development of smart agriculture.

## 5. Conclusions

To address the issues with traditional bitter gourd phenotype identification, which relies on time-consuming manual observation with low accuracy, this study introduces the ConvNeXt V2 module, SimAM attention mechanism, and WIoUv3 loss function to propose an improved network structure, CSW-YOLO. It achieved a detection precision of 94.6%, recall of 80.6%, mAP50 of 96.7%, and F1 score of 87.04%, marking improvements of 8.5%, 0.4%, 11.1%, and 4% over the previous YOLOv8, respectively. Additionally, heatmap analysis and ablation studies further validate the effectiveness of the network improvements, enhancing the model’s focus on relevant features, reducing the impact of irrelevant features on discrimination, and improving model generalizability and reducing false positive rates. Furthermore, compared to various mainstream deep learning models, CSW-YOLO’s performance is superior, and its FPS is sufficient to meet the needs in practical production. In summary, CSW-YOLO can more effectively handle the task of bitter gourd phenotype detection under various environmental backgrounds, accurately and efficiently extracting and utilizing the features of bitter gourd images, providing an efficient and precise detection technique for bitter gourd phenotypes. In the future, we will continue to optimize the target detection algorithm to improve the model’s accuracy and efficiency in complex scenarios, providing a solid technical foundation and rich practical experience for smart agriculture.

## Figures and Tables

**Table 1:** Descriptions of bitter gourd detection labels.

ID	Labels Name	Descriptions	Sample Size
1	R_Top_P_Tail	The top is rounded, the tail is pointed	264
2	R_Top_R_Tail	The top is rounded, the tail is blunt	241
3	R_Top_B_Tail	The top is rounded, the tail is round	482
4	R_Top_F_Tail	The top is rounded, the tail is flat	263
5	F_Top_P_Tail	The top is flat, the tail is pointed	224
6	F_Top_R_Tail	The top is flat, the tail is blunt	241
7	F_Top_B_Tail	The top is flat, the tail is round	411
8	F_Top_F_Tail	The top is flat, the tail is flat	221
9	P_Top_P_Tail	The top is pointed, the tail is pointed	212
10	P_Top_R_Tai	The top is pointed, the tail is blunt	249
11	P_Top_B_Tail	The top is pointed, the tail is round	285
12	P_Top_F_Tail	The top is pointed, the tail is flat	127

**Table 2:** Training results of various YOLO versions.

Models	Precision	Recall	mAP50	F1
YOLOv8n	86.10%	80.20%	85.60%	83.05%
yolov9	78.5%	62.9%	69.4%	69.84%
yolov10n	79.1%	65.8%	71.5%	71.84%
yolov11n	65.7%	64.2%	70.2%	64.94%

**Table 3:** Ablation study results.

ID	Backbone	Attention	Loss	Precision	Recall	mAP50	F1_Score
1	-	-	-	86.1%	80.2%	85.6%	83.05%
2	-	-	wiouv3	88.7%	85.5%	93.8%	87.07%
3	-	SimAM	-	89.9%	77.3%	93.6%	83.13%
4	ConvNeXt V2	-	-	93.5%	84.2%	97.2%	88.61%
5	-	SimAM	wiouv3	89.7%	82.2%	91.0%	85.79%
6	ConvNeXt V2	-	wiouv3	93.8%	92.1%	97.1%	92.94%
7	ConvNeXt V2	SimAM	-	94.0%	83.3%	96.3%	88.33%
8	ConvNeXt V2	SimAM	wiouv3	94.6%	80.6%	96.7%	87.04%

**Table 4:** Results of comparative experiments.

Models	Precision	Recall	mAP50	F1	FPS	Model Size/M
YOLOv7	88.8%	68.3%	80.9%	77.21%	72.57	71.4
YOLOv7-Tiny	49.6%	67.1%	56.2%	57.04%	82.30	11.7
YOLOv5	75.7%	88.7%	87.7%	81.69%	217.39	3.76
YOLOv5s	87.1%	65.5%	75.7%	74.77%	217.39	13.8
YOLOv3-Tiny	78.6%	64.0%	78.8%	70.55%	357.14	17.5
Faster-RCNN	75.8%	87.6%	91.3%	81.30%	18.53	108
YOLOv8n	86.1%	80.2%	85.6%	83.05%	144.93	5.96
YOLOv9m	74.7%	77.8%	80.7%	76.21%	122.26	32.4
YOLOv10s	60.9%	80.0%	75.2%	69.16%	258.19	15.8
YOLOv11s	75.5%	62.5%	79.9%	68.39%	255.31	18.3
CSW-YOLO	94.6%	80.6%	96.7%	87.04%	135.14	20.7

## Data Availability

Since the project presented in this research has not yet concluded, the experimental data will not be disclosed for the time being. Should readers require any supporting information, they may contact the corresponding author via email.
